# Global fingerprint of humans on the distribution of *Bartonella* bacteria in mammals

**DOI:** 10.1371/journal.pntd.0006865

**Published:** 2018-11-15

**Authors:** Hannah K. Frank, Scott D. Boyd, Elizabeth A. Hadly

**Affiliations:** 1 Department of Biology, Stanford University, Stanford, California, United States of America; 2 Department of Pathology, Stanford University, Stanford, California, United States of America; 3 Stanford Woods Institute for the Environment, Stanford University, Stanford, California, United States of America; 4 Center for Innovation in Global Health, Stanford University, Stanford, California, United States of America; University of California San Diego School of Medicine, UNITED STATES

## Abstract

As humans move and alter habitats, they change the disease risk for themselves, their commensal animals and wildlife. *Bartonella* bacteria are prevalent in mammals and cause numerous human infections. Understanding how this genus has evolved and switched hosts in the past can reveal how current patterns were established and identify potential mechanisms for future cross-species transmission. We analyzed patterns of *Bartonella* transmission and likely sources of spillover using the largest collection of *Bartonella gltA* genotypes assembled, including 67 new genotypes. This pathogenic genus likely originated as an environmental bacterium and insect commensal before infecting mammals. Rodents and domestic animals serve as the reservoirs or at least key proximate host for most *Bartonella* genotypes in humans. We also find evidence of exchange of *Bartonella* between phylogenetically distant domestic animals and wildlife, likely due to increased contact. Care should be taken to avoid contact between humans, domestic animals and wildlife to protect the health of all.

## Author summary

As humans move around the globe they contact new environments, potentially introducing novel diseases to wildlife, domestic animals and humans. Understanding how current infection patterns were established and how humans have likely altered them can help protect human, animal and environmental health. We traced the evolution of and distribution of globally distributed, pathogenic *Bartonella*, a common and well-studied bacterial genus in wildlife and humans that can cause cat scratch disease, trench fever and other diseases. We showed that humans are likely changing disease risk for themselves and the animals in their environment by moving themselves and domestic animals, as evidenced by large geographic movements of infections or shared infections in distantly related species. Not only does this increase our knowledge about *Bartonella*, an important emerging pathogen, but our investigation can serve as a model for elucidating the driving role of humans in changing disease landscapes.

## Introduction

Human movements and actions have numerous impacts for wildlife disease [1,2]. These impacts are of concern both from a wildlife conservation standpoint [1,2] and from a public health perspective (spillover). Over 60% of emerging infectious diseases in the world are zoonotic, meaning they are transmitted from animals to humans [3]. Despite the fact that zoonosis is an important component of emerging infectious diseases, it is often difficult to trace the ecology and evolution of zoonotic pathogens [4]. Most efforts to identify the source of zoonoses occur after a human has become infected. Because spillover events are rare and often infection prevalence in the reservoir species is low, it can be difficult to trace the origin of potential zoonoses. However, *Bartonella* bacteria are an exception to this pattern. This genus of bacteria has been found in numerous taxa and is usually at high prevalence [5]. *Bartonella* is a blood-borne pathogen, found in many animals. It is the cause of cat scratch fever, Carrion’s disease and trench fever as well as a number of incidents of endocarditis in humans and has been hypothesized to be the cause of unexplained febrile illness in a number of cases [5,6]. Therefore, it is an ideal pathogen to focus on in tracing zoonotic potential as well as potential impacts on the native hosts. In this study, we construct some of the largest global phylogenies to date of *Bartonella* from both 16s rRNA genes and citrate synthase (*gltA*) to determine the evolutionary history of *Bartonella*, patterns of host switching and geographic constraint and its spillover into humans or from human commensals into wild species. The citrate synthase gene is known to give high power to discriminate between *Bartonella* strains and is one of the most commonly sequenced *Bartonella* genes [7,8]. We also examine 16s as it is the most commonly sequenced locus for metagenomics studies, though it gives low power to discriminate between *Bartonella* species [8].

## Materials and methods

### Ethics statement

Research was approved by the Stanford University Administrative Panel on Laboratory Animal Care (protocol 26920) and conducted under the appropriate Costa Rican permits (RT-044-2015-OT-CONAGEBIO, RT-042-2015-OT-CONAGEBIO, 121-2012-SINAC, RT-019-2013-OT-CONAGEBIO, 226-2012-SINAC).

In order to ascertain broader patterns of spillover and *Bartonella* transmission between species, sequences were downloaded from Genbank on 30 November 2016 using the search term “Bartonella gltA” and again on 21 May 2018 using the search terms “Bartonella” AND (“gltA” OR “GltA” OR “GLTA” OR “glta” OR “citrate synthase”) and limiting the search to sequences uploaded in the previous 900 days in order to update our dataset with the most recently published sequences. A separate search was conducted using the search term “Bartonella 16s” on 1 February 2017. Insect microbiome studies that detected *Bartonella* were also used [9–15]. *Bartonella* from Costa Rican bats in a mosaic agricultural landscape, including previously published [16] and 67 new sequences (isolated as in Judson et al. [16]) are also incorporated in this study (Genbank accession numbers MH234314 –MH234380). Metadata were downloaded from Genbank and/ or confirmed by examining the cited publication and are summarized in S1 and S2 Files. When data in Genbank were not associated with a publication, geography was inferred by the host range and/or title information in Genbank. The host of questing ticks was undetermined and therefore denoted as “unknown.” In some cases, genomes of *Bartonella* strains were published independently from their hosts; in this case we searched other literature to find the source of the strain. Sequences that were not in fact *Bartonella* gltA were removed manually and sequences were aligned using the Geneious alignment algorithm and refined using MUSCLE in Geneious (version 8.1.9 [17]). Sequences that were significantly redundant (or multiple sequences of the same species of *Bartonella*) were excluded to reduce the size of the resultant phylogenies. This was especially the case for the published genomes of named *Bartonella* species; many of these sequences lacked data on when and where they were isolated and were therefore excluded from our analysis. We also excluded some fragments that were too short or had substantial missing data within the alignments, as well as fragments which misaligned significantly at the ends, causing us to doubt the quality of these end base calls. Sequences that contained one or two base pair deletions not found in other sequences were also eliminated as we doubted the quality of the sequences. Sequences with deletions in multiples of three base pairs were retained as these likely represent actual deletions of amino acids. Alignments were manually inspected and corrected. Two alignments were produced, one of 540 bp and one of 277 bp. The first contained 677 sequences in total and the second included 1,060 unique sequences.

In order to test for patterns in host specificity and biogeography we also constructed Bayesian phylogenies using BEAST 2 [18] for the 540 bp fragment and the 277 bp fragment. Alignments were split into three partitions based on the base pair’s position in the codon and run in PartitionFinder to determine the best nucleotide substitution models using AICc [19]. These parameters were then used to configure the parameters for the BEAST 2 run. For both the 540 bp and 277 bp run, PartitionFinder determined that all three positions should be run under the same mode, a GTR+I+G model. As empirical and maximum likelihood estimated base frequencies usually have little impact on the phylogeny, we used observed base frequencies for both sets of nucleotides [19].

We tested two different models for the phylogenetic hypothesis based on the 540 bp fragment. Both analyses were run with a gamma site model with empirical base frequencies, an estimated proportion of invariant sites and all nucleotide transition/transversion frequencies except the CT transition rate estimated. The gamma shape prior was set to an exponential distribution with a mean of 1; the proportion of invariant sites was set to a uniform distribution between 0 and 1; all nucleotide substitution rates were set to a gamma distribution with an alpha of 2 and a beta of 0.5 or 0.25 for transitions and transversions respectively. In all cases *Bartonella* was constrained to be monophyletic with *Brucella melitensis* as an outgroup. The first model tested was a strict clock model with a constant population size coalescent model with vague priors as has been used for previous phylogenetic analyses of *Bartonella* [20,21] with the population size prior set to a 1/X distribution. The second was a birth death model run with a log-normal distributed relaxed molecular clock. The birth rate prior was set to a uniform distribution between 0 and 10,000; the relaxed clock mean prior was set to a uniform distribution between negative infinity and infinity; the relaxed clock standard deviation prior was set to an exponential distribution with a mean of 1; the death rate prior was set to a uniform distribution between 0 and 1. Additionally a clade of *Artibeus lituratus* and *Artibeus watsoni*-associated *Bartonella* was estimated to have evolved at the divergence of the two bat species (KJ816682, MH234319, MH234329, MH234330) and the prior distribution was estimated with a log-normal distribution with a mean of 8.5 mya (SD = 2.73) based on previous estimates [22–27] collated in TimeTree [28]. We used this clade as a calibration point as it was strongly supported in all of our initial analyses, regardless of model and was nested within other Central American bat-associated strains and therefore unlikely to have been impacted by human influence.

For the 277 bp tree we ran our simulations with a GTR distribution, an estimated proportion of invariant sites and a gamma distribution of rates. We tested two models, a strict clock, constant population size coalescent model as described in the first model for the 540 bp alignment and a birth death model with a relaxed log normal clock as described in the second model for the 540 bp alignment. In both models we constrained *Brucella melitensis* to be an outgroup but no other calibrations were included. All *gltA* model were run for 2.5 x 10^7^ generations and sampled every 50,000 generations.

All *gltA* models converged with all parameters showing an effective sample size (ESS) over 100 (with the exception of the inferred relative death rate in the relaxed clock model of the 540bp alignment) and most showing an ESS over 200. The two models for the 540bp alignment were compared using AICM of the likelihood [29] with 1,000 bootstraps implemented through Tracer as model comparison using path sampling was not practical. For the 540bp alignment the best model was the second–a relaxed log normal clock calibrated with host divergence dates (dAICM = 356.48). For the 277bp alignment a strict clock was favored over a relaxed clock (dAICM = 441.86). Maximum clade credibility (MCC) trees were produced using TreeAnnotator, mean heights and a burn in of 10%.

In order to understand the evolutionary origin of *Bartonella* we constructed a phylogeny using sequences from the 16s rRNA gene. All 450 sequences were aligned and trimmed to the same length (259 bp) in Geneious. We constructed a phylogenetic hypothesis in BEAST 2 using a strict clock and a birth death model with vague priors as described in the birth death models for the *gltA* genes with *Rhizobium leguminosarum* as an outgroup. The model was run for 10^7^ generations; most ESS were above 300, though the birth rate and death rate ESS were roughly 100. As we were not concerned with speciation dynamics but rather broad topology, we considered this hypothesis to be sufficiently sampled.

The 277 bp *gltA* MCC tree was used in an analysis of host specificity and geographic conservation between related *Bartonella* species. Many nodes did not have good support so we conducted all analyses using only nodes with a Bayesian posterior probability of the likelihood of 0.7 or above. Using the fitDiscrete function in geiger [30], four models of discrete character evolution were fit—one using a lambda transformation, one using a white noise transformation, one using an early burst transformation and one using no transformation to model the evolution of host order (with strains isolated from ectoparasites assigned to the ectoparasite’s host) and broad geographic region of isolation both by continent (all except Antarctica) and by Old World versus New World. Fit of the models was assessed using AICc weights and log-likelihoods.

Host switches and sharing of clades between geographic regions was assessed by manually examining the MCC phylogenetic hypothesis based on a 277bp fragment of *gltA*, by examining the location of Genbank records with identical genotypes and by searching the literature for the distribution of named *Bartonella* species. A host switch or geographic shift was inferred so as to capture the minimum number of shifts with Bayesian posterior probabilities of at least 0.7. We also assessed shifts at posterior probabilities of the likelihood of at least 0.9 and 0.95 to ensure our results were robust regardless of our cut-off.

In inferring the influence of humans on *Bartonella*, we categorized genotypes or monophyletic clades as being found in one or more of the following categories: rats, other rodents (excluding rats), humans, domestic carnivores, wild carnivores, domestic artiodactyls, wild artiodactyls, shrews, and bats. We also noted other wild animals that were rarer in our dataset (e.g. pikas, wild hares and wild primates). Genotypes found in rats, domestic animals and humans were categorized as “human-associated” and the rest as “non-human-associated”. Human-associated *Bartonella* in this study does not necessarily mean the genotype has been found in a human but rather in a human or a commensal animal or an ectoparasite on a human or commensal animal. Sometimes the metadata contained within Genbank and the publications were insufficient to allow us to distinguish the exact species from which the *Bartonella* was isolated. All genotypes isolated from *Rattus* sp., their ectoparasites or an organism denoted “rat” were counted as human-associated rats. Although many species of rodents are commensal with humans (e.g. [31]), all other rodents were counted as not being human-associated to avoid the need to categorize over 100 rodent species, as well as incorporate rodents for which the species was unknown (N = 50). This division therefore renders our analyses of human-associated strains conservative.

Additionally, we determined all instances of *Bartonella* transferring between host orders represented in our dated, 540bp phylogenetic hypothesis to determine the ages of such transfers and test whether transfers to humans were more recent than other transfers. For these analyses we only used clades with at least 0.7 posterior probability support, which meant that a few (3) clades were assessed as older than they may actually be. In each of these cases the transfer involved a genotype found in a human, meaning our hypothesis testing is conservative. Difference between zoonotic transfers and other transfers was assessed using one-tailed t-tests assuming unequal variance on the inferred node age and lower bound of the 95% highest probability density to account for uncertainty in dating of the nodes.

All alignments, metadata and R code are available in the supporting information.

## Results

Starting with 2,564 *gltA* sequences, we analyzed 2,515 277 bp sequences, 1,060 of which were unique and used to construct a phylogenetic hypothesis. Information on identical sequences used to infer host and geography transfers is included in S3 File. In our final dataset, the most commonly sampled taxa were rodents (N = 1,143 of which 94 were rats) and bats (N = 374). We were also able to align a 540bp *gltA* fragment for 677 genotypes, a subset of the larger dataset.

### Evolutionary history

**Fig 1:**
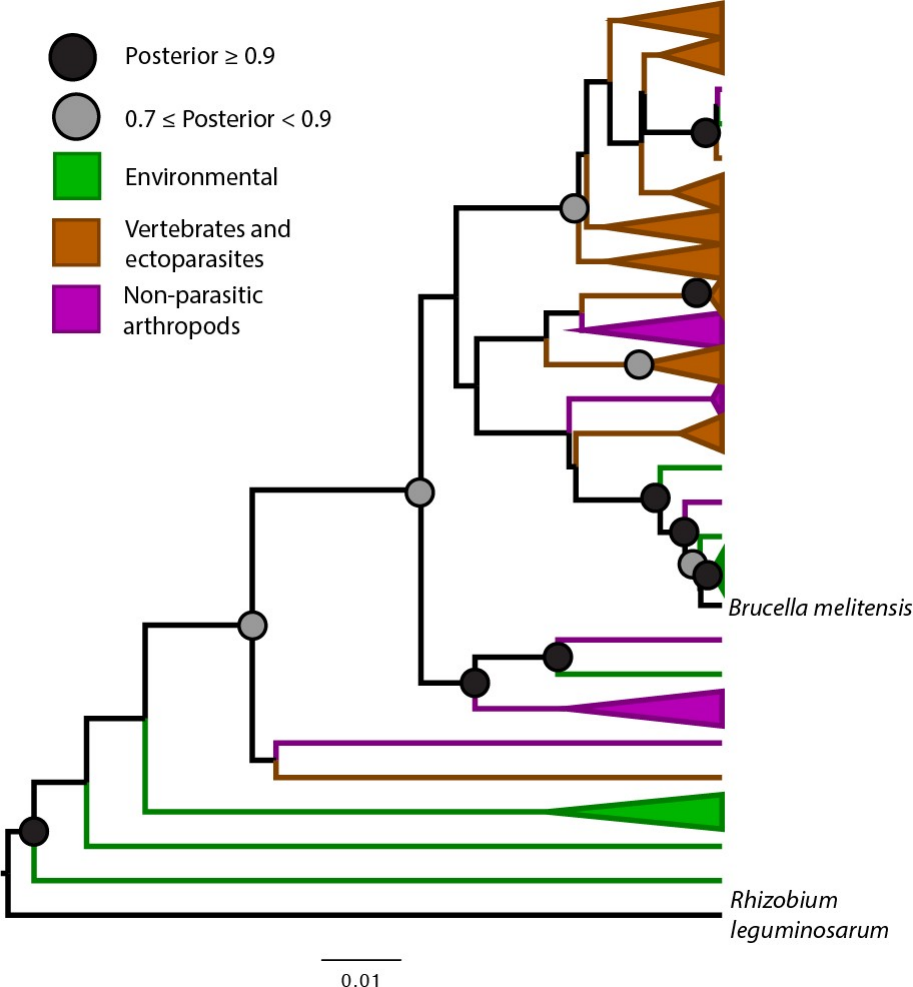
Bayesian phylogenetic hypothesis of *Bartonella* genotypes based on a 259bp fragment of 16s rRNA gene. Ectoparasites and their vertebrate hosts are colored brown; environmental sequences are green; non-ectoparasitic arthropods are colored purple. Scale bar indicates substitutions per site. “Posterior” refers to the Bayesian posterior probability of the likelihood.

**Fig 2:**
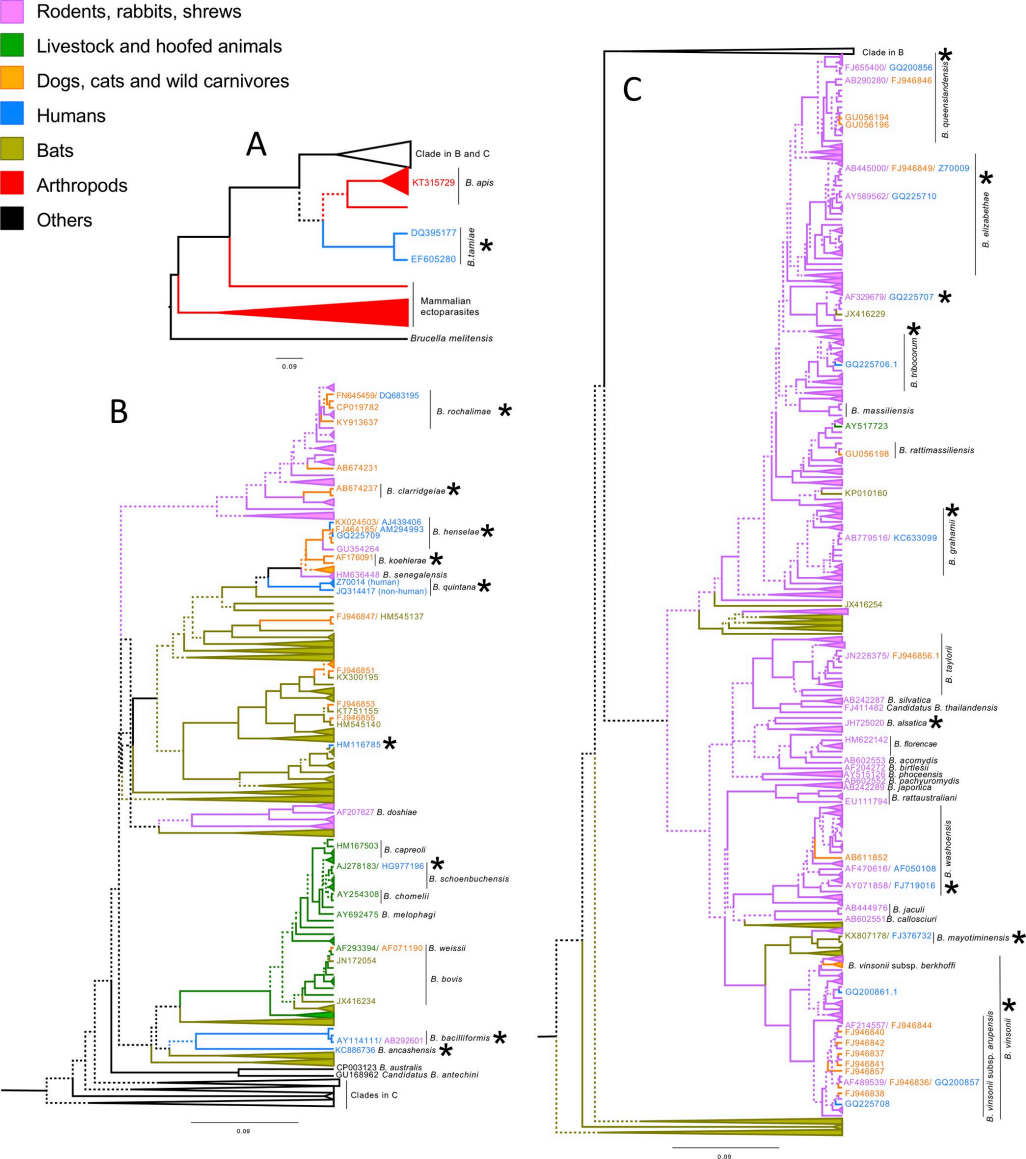
Bayesian phylogenetic hypothesis of *Bartonella* genotypes based on a 277 bp fragment *gltA*. Tip labels and branches have been colored according to the taxa in which they were identified with ectoparasites colored according to their host and collapsed to highlight specific patterns. For readability, basal clades are shown in A, a subset of the more derived clades is shown in B, and the rest of the clades are shown in C. Branches with less than 0.7 Bayesian posterior probability of the likelihood support (indicating greater uncertainty) are dashed. Accession numbers separated by slashes indicate identical genotypes that were isolated from different hosts. Asterisks indicate strains found in humans, whether zoonotic or human-hosted strains.

Phylogenetic hypotheses generated from a 277 bp fragment of *gltA* and a 259 bp fragment of the 16s rRNA gene both support an origin for *Bartonella* in the environment and in the guts of insects (both ectoparasitic and non-ectoparasitic species; Figs 1 and 2). Twice *Bartonella* has infected mammals from these environmental samples, which are basal to the main clade of mammal-associated *Bartonella* (Fig 2), which likely invaded mammals approximately 79 million years ago based on our time calibrated phylogeny, though it is unclear which mammalian host is ancestral (Figs 2 and S1).

### Host and geographic conservation

*Bartonella* are generally highly host-specific with closely related genotypes found in the same order of host; the best model of evolution for *Bartonella* host order in the 1,039 analyzed 277bp fragments (excluding basal mammalian ectoparasites, rare host orders and taxa for which host order was unknown) was a lambda model in which lambda was 0.96, indicating near Brownian motion evolution along the phylogeny (AICc weight = 1.00; S1 Table). Similarly, in an analysis of 1,027 fragments, closely related genotypes of *Bartonella* were generally in the same geographic regions, whether analyzed by the continent from which the genotype was isolated or Old World versus New World (best model for both was a lambda transformation; continent: λ = 0.98, AICc weight = 1.00; OW-NW: λ = 0.97; AICc weight = 1.00; S2 and S3 Tables). Basal strains of *Bartonella* from arthropods were excluded from these analyses as they appeared to be widely distributed, environmental isolates that could skew the analyses.

### Exceptions to host specificity and limited geographic range: Zoonosis and the human-domestic-wildlife interface

Despite the overall high host specificity of *Bartonella*, we observed a number of host shifts in our large phylogenetic hypothesis (S4 File and S2 Fig). Of 19 spillovers into humans (Figs 2 and S2 and S3 and S4 Files; S4 Table), seven were from rodent clades (GQ200856, Z70009, GQ225707, GQ225706, KC633099, AF050108, GQ200861). In some cases, multiple strains of the same *Bartonella* species have infected humans representing separate spillovers, such as in the case of *B*. *washoensis* or the *B*. *vinsonii* complex which is found in both rodents and canids [32]. Two human infections appear to stem from bats (*B*. *mayotimonensis* [33] and HM116785, found in a Polish forest worker [34] and most closely related to European bat ectoparasite-associated genotypes), one from rabbits (*B*. *alsatica*), one from roe deer (HG977196) and four from cats and dogs (*B*. *koehlerae*, *B*. *clarridgeiae*, *B*. *rochalimae*, *B*. *henselae* [32]). Four genotypes were of uncertain origin–*B*. *tamiae*, a basal infectious strain that causes febrile illness in humans in Asia and has been found in rodent ectoparasites [35,36]; *B*. *bacilliformis*, a causative agent of Carrion’s disease and verruga peruana [37]; *B*. *ancashensis*, a causative agent of verruga peruana [38] and *B*. *quintana*, the causative agent of trench fever. *Bartonella quintana* has also been found in fleas on gerbils [39] and grouped with Old World rodent and bat-associated genotypes, as well as *B*. *koehlerae* and *B*. *henselae*. Both *B*. *bacilliformis* and *B*. *ancashensis* are known primarily from humans [40]; *B*. *quintana* is found in both humans and macaques but the genotypes found in macaque are diverged from human *B*. *quintana* (95% HPD: 277 ka-5.7 mya; S1 Fig and S5 File).

Rodent-hosted *Bartonella* has infected domestic carnivores seven times (dogs and cats; representative genotypes: GU056194, FJ946849, GU056198, AF148486, FJ946842, FJ946856, FJ946846) and artiodactyls (a cow ectoparasite; AY517723) once; in five instances rodents and domestic carnivores shared *Bartonella* but the direction of transmission could not be inferred (representative genotypes: CP019782, EF616739, HM636448, AF214557, AF489539) and in two instances *Bartonella* from domestic animals has infected rodents (GU354264, GU573930). Bats and rodents have exchanged *Bartonella* at least five times (representative sequences: EU167549, KP010160, MH234340, AB779518, AF148493). Bats and domestic animals have exchanged *Bartonella* six times; in four cases bat-associated *Bartonella* has infected dogs (representative sequences: KP100359, KX300195, HM545140, KT51155); in one case *B*. *bovis* infected a bat ectoparasite (JN172054) and in another case *Bartonella* from a dog may have infected a bat (FJ946847/ HM545137).

In eight cases, there was a transfer of *Bartonella* between domestic and wild animals (S4 File). In three cases *Bartonella* associated with domestic carnivores infected wild carnivores. Domestic cat-associated *Bartonella* has been found in lions (KX499327), mongooses (MF959421) and a cheetah (KX499331); domestic dog-associated *Bartonella* (*B*. *vinsonii berkhoffii*) has infected a fox (KU292568). Additionally, *B*. *rochalimae* has been found in a number of wild canids such as coyotes and foxes and has infected domestic dogs. And *Bartonella* has been shared between (1) a skunk, raccoon, cats and dogs (e.g. CP019782/ CP019786) and (2) domestic cats, lynx, mountain lions, cheetahs and lions (e.g. KX499327/ KX024503). Additionally, in at least two cases *Bartonella* transferred between domestic artiodactyls and wild artiodactyls–in one case a clade included roe deer, elk, cattle and sheep; in the other *B*. *bovis* was found in elk (KB915625).

We also inferred nine transfers of *Bartonella* between rodents and shrews, which are phylogenetically quite distant but presumably share the same terrestrial habitats and some of the same ectoparasite vectors (S4 File). Similarly, we inferred four transfers of *Bartonella* between rodents and lagomorphs. Other transfers included *B*. *bovis* infecting a cat (*Bartonella weissi*, AF071190); a *Bartonella* strain found in a Japanese marten (AB611852) and one in a raccoon (KU292571) in clades of rodent-associated *Bartonella*; the evolutionary divergence of *Bartonella* in *Antechinus* and kangaroos; the divergence of human and non-human primate *B*. *quintana* and the grouping of artiodactyl-associated *Bartonella* with strains found in bats.

We inferred 64 instances where *Bartonella* was transferred between orders or within orders between wild and domestic animals (S4 File); 42 of these instances were associated with domestic animals, rats or humans, while 21 were not and were presumed to have occurred naturally (including the evolutionary divergence between human and non-human primate *B*. *quintana*) and 1 instance involved domestic animals, rats and humans as well as other rodents and shrews. Nearly twice as many instances of host shifts were associated with human influence than natural transitions.

Additionally, we noted 118 instances in which monophyletic clades or single genotypes contained genotypes isolated from more than one continent/ geographic region, 56 of which spanned both the Old World and New World, denoted in parentheses (S4 File and S2 Fig). Of the clades, 55 (27) involved genotypes found in non-rat rodents, 27 (16) involved rats, 12 (11) involved humans, 26 (16) involved cats and dogs, 4 (0) involved domestic hoof stock, 8 (5) involved wild carnivores, 8 (7) involved shrews, 4 (2) involved pikas and hares, 2 (1) involved wild artiodactyls and 38 (7) involved bats. When we analyzed only clades found on more than one continent and grouped humans, cats, dogs, rats and domestic artiodactyls together as human-associated strains, the human-associated strains were more likely to be distributed across both the Old World and New World than other strains (Fisher’s exact test, p = 0.0027, Odds ratio = 3.25). This result was robust regardless of our posterior cut off criteria (0.9 support or above: p = 0.0047, Odds ratio = 3.35; 0.95 support or above: p = 0.013, Odds ratio = 2.98).

Transfers of *Bartonella* into humans were also more recent than other divergences we inferred based on both the estimated age of the node (mean_zoonotic_ = 4.56 my; mean_non-zoonotic_ = 12.52 my; t-test: t = -2.24, df = 20.9, p = 0.018) and the lower bound of the 95% highest posterior density (mean_zoonotic_ = 0.656 my; mean_non-zoonotic_ = 2.485 my; t-test: t = -2.43, df = 21.8, p = 0.012; S5 File).

## Discussion

Our phylogenetic hypothesis based on the 277bp fragment of *gltA* was broadly concordant with that of other studies of *Bartonella*. We recovered a monophyletic grouping of *B*. *grahamii*, *B*. *rattimasiliensis*, *B*. *tribocorum*, *B*. *elizabethae* and *B*. *queenslandensis*; a monophyletic grouping of *B*. *quintana*, *B*. *henselae* and *B*. *koehlerae*; and the clustering of *B*. *vinsonii* subspecies consistent with studies from multiple *Bartonella* genes [6,41]. These groupings are further supported by a large 509 gene phylogeny, which also supported our findings of basal positions for *B*. *apis* and *B*. *tamiae* and monophyletic grouping of artiodactyl-associated *Bartonella* [42]. In fact, most of our deeper branching relationships were also consistent with this large, multigene phylogeny, though were significantly less well supported as our conclusions were based on a single gene.

### *Bartonella* as an environmental bacterium turned insect gut symbiont turned vertebrate pathogen

The proliferation of studies investigating *Bartonella* in various wildlife populations allows for greater insights into the origins and evolution of *Bartonella* and its potential for spillover more than ever before. Bartonellaceae is nested within the Rhizobiales, a lineage of soil bacteria that contains nitrogen-fixing root-associated members [43]. In our study, the most basal strains of *Bartonella* were found in environmental samples and arthropods (Figs 1 and 2). Additionally, gut microbiome studies from a variety of insects have revealed that *Bartonella* is actually widespread across arthropods, occurring in carrion beetles, butterflies, bees, various species of ants and a wide variety of ectoparasitic species [9–15]. Other studies have hypothesized that perhaps *Bartonella* may have a commensal role in the arthropods that vector it [44,45]. This led us to hypothesize that *Bartonella* originated as an environmental bacterium that was picked up by arthropods in which it diversified.

Because most metagenomic studies of bacteria amplify the 16s rRNA gene, there is a large amount of 16s data available and also *Bartonella* can be detected in samples that would not *a priori* be hypothesized to contain *Bartonella*, such as non-hematophagous insects or environmental samples. We mined GenBank for *Bartonella* 16s sequences to test our hypothesis that *Bartonella* is an environmental bacterium that became an insect commensal before becoming a vertebrate pathogen. The 16s rRNA gene is much less powerful for discriminating *Bartonella* species than *gltA* [7] and often metagenomic studies amplify only very small fragments of the gene, making it difficult for us to resolve fine scale diversification but we were able to determine that basal strains of *Bartonella* were largely found in environmental samples and non-hematophagous insects (Fig 1). Additionally, work on *Bartonella* has shown that the evolution of a type 4 secretion system, along with selection on other invasion mechanisms [43], has been instrumental in allowing *Bartonella* to diversify and invade host cells [46,47] while other work has shown *Bartonella* can incorporate a type 4 secretion system via lateral gene transfer when it coinfects an amoeba with *Rhizobium radiobacter* [48]. Further, examinations of lateral gene transfer of metabolic genes in *Bartonella* reveals that many of these genes derive from common insect gut commensal bacteria [49]. We strengthen the suggestions of these previous studies by drawing data from insect and environmental metabarcoding studies and demonstrating their basal phylogenetic position within *Bartonella*.

### *Bartonella* spillover is predominantly from rodent and domestic animals

Using the literature [32] and isolates from published sequences on GenBank, we identified 19 genotypes of *Bartonella* that have been detected in humans (most of which are also known to cause disease; S4 Table). Of these, eight of the genotypes are most closely related to genotypes found in rodents and four are distributed in dogs and cats but have spilled over into humans. *Bartonella vinsonii* forms a species complex that is associated with dogs (subsp. *berkhoffi*) and rodents (subsp. *arupensis*). We inferred at least three separate transfers of *B*. *vinsonii* (two instances of *B*. *v*. *arupensis* and one instance of *B*. *v*. *vinsonii*) from rodents into humans based on phylogenetic relationships (GQ200861, GQ200857, GQ225708), confirming previous conclusions [6]; however, we treated these as a single spillover for the sake of simplicity. Additionally, we identified a genotype of *Bartonella* detected in a febrile patient in Thailand (GQ200856) as having over 95% identity with *B*. *queenslandensis*, a genotype first found in Australian rodents and also found in numerous Asian rodents, suggesting a previously unappreciated possible rodent-human transmission. Interestingly, one *Bartonella* genotype that was recovered from a Polish forest worker (HM116785) most closely resembled genotypes found in European *Myotis*, a genus of bat, and their ectoparasites (JQ695834, JQ695839, KR822802). That most strains isolated from humans are related to domestic or peridomestic animals strongly indicates that spillover of *Bartonella* requires close contact between humans and the natural reservoirs of these infectious strains.

However, when examined at a broader scale, many of these genotypes are related to genotypes found in wild animals. For example, *B*. *henselae*, *B*. *koehlerae* and *B*. *quintana* were closely related to *Bartonella* detected in African rodent ectoparasites and an Asian bat. Similarly, *B*. *mayotimonensis* was closely associated with genotypes of Central American bats detected in this study. This same isolate has also been found in bats in Europe [33,50] and most recently North America [51]. This suggests that bats may be a possible reservoir of potentially zoonotic *Bartonella* strains but that infrequent contact between bats and people prevents transmission. Rather most of the transmission we infer requires the transmission of *Bartonella* into a domestic or peridomestic animal, which can then transmit it to humans. Despite the noted host specificity of *Bartonella* (S1 Table), the diversity of strains that infect humans and their distribution across the phylogenetic tree of *Bartonella* suggests that this bacterial genus can and will switch hosts when given the opportunity (especially when hosts are immunocompromised [52,53]). The relative evolutionary lability of these genotypes is further underscored by the instances in the global phylogeny of genotypes being exchanged between bats and rodents (at least five times; S4 File).

Overall, we found that rodents were responsible for more transmission of *Bartonella* into humans than any other group, followed by domestic carnivores. Rodents also transmitted the most *Bartonella* to domestic animals and bats, though infections likely originating from wildlife such as bats in domestic animals are also relatively common. One potential explanation for the prominence of rodents in host switching may be the generalist tendencies of their ectoparasites. *Bartonella* is vectored by arthropods but some ectoparasites, such as blood sucking hippoboscid flies, are very highly host specific [54] potentially preventing cross-species transmission. In contrast, many rodents host fleas which can bite other taxa and have been found to host many genotypes of *Bartonella* that have originated in rodents and infected other species such as humans (e.g. [55,56]). Considerations of the host specificity of the vector species may be very important for determining the risk for disease spillover and indeed public health officials recommend avoidance of potential vectors as the most important measure for prevention of bartonellosis [32].

It is important to note, however, the constraints on our conclusions due to available data. We only have a small fragment of *gltA* to examine across these 1,060 genotypes, making inferences at deep nodes uncertain and potentially artificially grouping together isolates that are identical at the sites we examined but that may differ dramatically at other important genetic loci or even other loci within the *gltA* gene. Additionally, we are limited to the animals that have been sampled, which are overwhelmingly bats and rodents, as well as symptomatic humans. It is possible and highly likely that there are animal intermediates between these transmission events that are missing, which obscures our ability to infer the directionality of transfer. Indeed, to our knowledge, direct *Bartonella* transmission has not been observed between bats and domestic animals; however, we observed multiple instances in which *Bartonella* detected in domestic animals and their ectoparasites fell evolutionarily within *Bartonella* detected in bats. This suggests that these strains may have originated in bats before being transmitted to domestic animals and demonstrates the power of a large-scale phylogenetic approach to identify unlikely sources of *Bartonella* transfer.

### Human movements shape *Bartonella* diversification and infection patterns

Another interesting pattern that emerged when examining the tree as a whole was the impact of humans in spreading *Bartonella* strains and infections globally. A few particular mammalian species that are associated with humans, such as dogs, cats, cows and rats, have managed to bring their strains of *Bartonella* globally [21,32,47,57–61]. Rats, in particular the genus *Rattus*, were very common in the largest clade of globally distributed rodent *Bartonella* (the clade containing *B*. *queenslandensis*, *B*. *elizabethae*, *B*. *tribocorum*, *B*. *massiliensis*, *B*. *rattimassiliensis* and *B*. *grahamii*), with representatives on nearly every continent. This clade also contains at least five zoonotic genotypes of *Bartonella*, as well as genotypes found in dogs and ectoparasites on dogs, bats and a cow, underscoring the important role of human commensals in spreading disease to humans, domestic animals and wildlife and across the globe.

There was a lot of uncertainty in the dating of our divergence times (in one instance three identical genotypes were inferred to be over 700,000 years diverged) perhaps due to the small fragment we were able to analyze and the depth of evolutionary history we were exploring. Additionally, there are many genotypes that may have died out or have not been sampled that mean even our minimum divergence date estimates are likely conservative. We cannot therefore state with certainty that humans are responsible for moving other species around, changing disease risk for themselves and wild animals. However, the finding that strains associated with humans or their domestic animals were more likely to be found globally, the finding that transfers between humans and other groups were the most recent ones, and the diverse placement of human infections across the phylogeny strongly support a role for humans changing their disease risk as they insert themselves and their associated animals into new habitats and ecosystems.

Such movements and increased contact between humans, domestic animals and wildlife not only disguise geographic patterns of *Bartonella* diversification (e.g. *B*. *queenslandensis*, first described in Australia [62], in *Rattus norvegicus* in Louisiana (AF075162; [57])) but have also led to presumably novel sharing of *Bartonella* between introduced domestic and peridomestic animals and native wildlife. For example, identical genotypes were found in a Chinese *Rattus* individual (DQ986952) and a white-footed mouse, a North American native (AY064534). If the introduced bacteria have adverse fitness consequences, this could be another human-mediated conservation concern. Domestic animals also present a health risk to wild animals and other domestic animals; *B*. *bovis* was found in a cat, an elk and a bat ectoparasite and *Bartonella* has been transferred between Old World bats and dogs. The pet trade exacerbates this by shipping exotic animals all over the world, changing the pool of available infections for both the introduced and native species [63]. Introduction of domestic species is causing sharing between these species and wild species, changing the disease risks for both.

Overall our findings show that *Bartonella* is a rich system for examining the impacts of humans on patterns of infectious disease spread within species and between species, across landscapes and across the globe. Phylogenetic inferences about the origin of infections should be interpreted with caution as they are heavily influenced by available data and the taxa that have been sampled. There may be many missing links between those we inferred but the hosts simply have not been sampled. At least some part of the noted host specificity of *Bartonella* seems to be due to ecological factors regulating exposure rather than immunological incompatibility. Given the diversity of sources of zoonotic strains, including divergent strains with similar clinical presentations, physicians and researchers should consider a broad range of potential animal hosts and screen for a wide range of *Bartonella* genotypes when investigating the source of a suspected *Bartonella* infection.

New sequences generated as part of this study have been uploaded to Genbank (accession numbers MH234314 –MH234380).

## Supporting information

S1 Table Model summaries for evolution of *Bartonella* host order. (DOCX)

S2 Table Model summaries for evolution of *Bartonella* geography (Continent). (DOCX)

S3 Table Model summaries for evolution of *Bartonella* geography (Old World versus New World). (DOCX)

S4 Table Reservoirs, phylogenetic context and geographic regions of *Bartonella* genotypes found in humans. Reservoir and geographic data are derived from *gltA* metadata, Breitschwerdt [32] and cited references. Phylogenetic context refers to placement in both the 277bp and 540bp MCC trees.
(DOCX)

S1 Fig Maximum clade credibility phylogeny based on 540 bp fragment of *gltA* with estimated branch timing. The numbers in brackets indicate the lower and upper bounds of the 95% highest posterior density of the height of that branch, an estimate of the age of divergence of the node that the branch leads to. The width of the circle at each node is proportional to the Bayesian posterior probability support at that node. The blue tick mark numbers indicate the well-supported instances of *Bartonella* transferring between hosts that are used in inferring the timing of host transfers as detailed in S5 File.
(PDF)

S2 Fig Maximum clade credibility phylogeny based on 277 bp fragment of *gltA* illustrating the order of the host and geographic region from which *Bartonella* genotypes were isolated. The color of the tip label indicates the order of the host from which the genotype was derived (ectoparasites are colored according to their host). Highlighted tips indicate that multiple samples had the same sequence and the duplicate sequences can be found in S3 File by searching for the Genbank accession number. The number on each branch indicates the Bayesian posterior probability support leading to the node on the right. The icon at each tip indicates the geographic region from which the genotype was derived and the colored dot at each tip indicates the type of host it was isolate from, e.g. *Rattus* sp. rodents, non-*Rattus* rodents, wild animals, domestic animals. “SM” indicates a shrew or rodent (small mammal) as sometimes the actual host was not given and “Unk” indicates the continent from which the genotype was isolated is unknown. If a *Bartonella* species name is listed at a tip but not a Genbank accession number, the accession number is that of the sequence used in Judson et al. [16].
(PDF)

S1 File Metadata for all *gltA* sequences. (XLSX)

S2 File Metadata for all 16s sequences. (XLSX)

S3 File Groups of identical sequences in 277 bp *gltA* analysis. Only one representative per group is included in the phylogeny and S2 Fig even though identical genotypes may have been found in other hosts or geographic regions. This file lists all Genbank accession numbers for sequences found in more than one individual in the study. A legend for all categories appears on the second sheet of the file.
(XLSX)

S4 File Summary of observed host and geographic transfers. This file lists every inferred host transfer or finding of a genotype or monophyletic clade on more than one continent. A legend for interpreting each category appears on the second sheet of the file.
(XLSX)

S5 File Inferred divergence times of host switches based on 540bp tree. This file lists every well-supported host switching event captured in our 540bp phylogenetic hypothesis. A legend for interpreting each column appears on the second sheet of the file.
(XLSX)

S6 File 277 bp *gltA* alignment. (NEX)

S7 File 540 bp *gltA* alignment. (NEX)

S8 File 259 bp 16s alignment. (NEX)

S9 File Final 277bp *gltA* maximum clade credibility phylogenetic hypothesis. (TREES)

S10 File Final 540bp *gltA* maximum clade credibility phylogenetic hypothesis. (TREES)

S11 File Final 16s maximum clade credibility phylogenetic hypothesis. (TREES)

S12 File R code for evolutionary model fits on the 277bp tree. (R)
