# Supplementary material for: Global fingerprint of humans on the distribution of Bartonella bacteria in mammals
Source: PLoS Negl Trop Dis. 2018 Nov 15;12(11):e0006865. doi: 10.1371/journal.pntd.0006865 (PMC6237287; doi:10.1371/journal.pntd.0006865)
Supplement: S1 Table — (DOCX) [file pntd.0006865.s001.docx]

**S1 Table**: Model summaries for evolution of *Bartonella* host order

| Model | Parameters | Log-likelihood | AICc | Delta AICc | AICc weight |
| --- | --- | --- | --- | --- | --- |
| **Lambda** | **λ = 0.96** | **-547.754** | **1099.52** | **0** | **1.00** |
| White Noise |  | -1178.224 | 2368.53 | 1269.01 | 0.00 |
| Early Burst | a = 1.60 | -563.179 | 1130.37 | 30.85 | < 0.0001 |
| None |  | -563.518 | 1129.04 | 29.52 | <0.0001 |
