# Supplementary material for: Global fingerprint of humans on the distribution of Bartonella bacteria in mammals
Source: PLoS Negl Trop Dis. 2018 Nov 15;12(11):e0006865. doi: 10.1371/journal.pntd.0006865 (PMC6237287; doi:10.1371/journal.pntd.0006865)
Supplement: S2 Table — (DOCX) [file pntd.0006865.s002.docx]

S2 Table: Model summaries for evolution of *Bartonella* geography (Continent)

| Model | Parameters | Log-likelihood | AICc | Delta AICc | AICc weight |
| --- | --- | --- | --- | --- | --- |
| **Lambda** | **λ = 0.98** | **-848.271** | **1700.55** | **0** | **0.996** |
| White Noise |  | -1516.374 | 3042.81 | 1342.25 | 0.00 |
| Early Burst | a = 6.05 | -853.72 | 1711.45 | 10.90 | 0.004 |
| None |  | -859.355 | 1720.72 | 20.16 | <0.001 |
