# Supplementary material for: Global fingerprint of humans on the distribution of Bartonella bacteria in mammals
Source: PLoS Negl Trop Dis. 2018 Nov 15;12(11):e0006865. doi: 10.1371/journal.pntd.0006865 (PMC6237287; doi:10.1371/journal.pntd.0006865)
Supplement: S3 Table — (DOCX) [file pntd.0006865.s003.docx]

Table S3: Model summaries for evolution of *Bartonella* geography (Old World versus New World)

| Model | Parameters | Log-likelihood | AICc | Delta AICc | AICc weight |
| --- | --- | --- | --- | --- | --- |
| **Lambda** | **λ = 0.97** | **- 281.1** | **566.3** | **0** | **0.997** |
| White Noise |  | -700.4 | 1402.8 | 836.6 | 0.00 |
| Early Burst | a = 2.2 | -287.9 | 580.0 | 13.8 | < 0.01 |
| None |  | -299.3 | 578.6 | 12.4 | < 0.01 |
