# Supplementary material for: Global fingerprint of humans on the distribution of Bartonella bacteria in mammals
Source: PLoS Negl Trop Dis. 2018 Nov 15;12(11):e0006865. doi: 10.1371/journal.pntd.0006865 (PMC6237287; doi:10.1371/journal.pntd.0006865)
Supplement: S4 Table — Reservoir and geographic data are derived from gltA metadata, Breitschwerdt [32] and cited references. Phylogenetic context refers to placement in both the 277bp and 540bp MCC trees. (DOCX) [file pntd.0006865.s004.docx]

S4 Table. Reservoirs, phylogenetic context and geographic regions of *Bartonella* genotypes found in humans. Reservoir and geographic data are derived from *gltA* metadata, Breitschwerdt [1] and cited references. Phylogenetic context refers to placement in both the 277bp and 540bp MCC trees.

| Human isolate | Reservoir | Phylogenetic context | Geography |
| --- | --- | --- | --- |
| *B. alsatica* | Rabbits | Likely in a large clade with *B. taylorii*, *B. washoensis* and *B. vinsonii*, globally distributed rodent and Central American bat-associated genotypes | Europe |
| *B. ancashensis* | Unknown | Basal mammalian polytomy | South America [2] |
| *B. bacilliformis* | Humans | Basal mammalian polytomy | South America |
| *B. clarridgeiae* | Cats | Related to North American rodent-associated genotypes and *B. rochalimae* | North America, Europe, South America, Asia |
| *B. elizabethae* | Rats (*Rattus*) | Globally distributed rodent-associated clade including *B. queenslandensis*, *B. massilensis, B. tribocorum, B. rattimassilensis, B. grahamii* | Asia, Africa, North America, Europe |
| *B. grahamii* | Rodents | Globally distributed rodent-associated clade including *B. queenslandensis*, *B. massilensis, B. tribocorum, B. rattimassilensis, B. elizabethae* | Asia, Europe, North America |
| *B. henselae* | Cats, Dogs | Sister to *B. koehlerae*, also associated with Old World rodent ectoparasite and bat associated genotypes and *B. quintana* | Asia, South America, Africa, North America, Europe, Australia [3] |
| *B. koehlerae* | Cats, Gerbils | Sister to *B. henselae*, also associated with Old World rodent ectoparasite and bat associated genotypes and *B. quintana* | North America, Europe, Africa, South America |
| *B. mayotiminensis* (FJ376732) | Bats [4,5] | North American bat and North American and Asian rodent – associated genotypes | Person in Iowa [6], North and Central America |
| *B. queenslandensis* | Rats (*Rattus*) | Globally distributed rodent-associated clade including *B. elizabethae*, *B. massilensis, B. tribocorum, B. rattimassilensis, B. grahamii* | Australia predominantly, Asia, North America |
| *B. quintana* | Humans | Old World rodent ectoparasite and bat associated genotypes; *B. henselae*; *B. koehlerae* | Europe, North America, Asia, Africa [7], Australia [8] |
| *B. rochalimae* | Dogs, Rodents | European rodent ectoparasite-associated genotype; Asian and North American rodent associated - genotypes | Traveler to Peru [9], North America, Europe, South America, Asia, Africa |
| *B. schoenbuchensis* | Roe deer, elk | Old World artiodactyl – associated genotypes | Europe |
| *B. tamiae* | Rats (*Rattus*) [10] | Basal to other mammalian *Bartonella* | Southeast Asia |
| *B. tribocorum* | Rodents | Globally distributed rodent-associated clade including *B. elizabethae*, *B. massilensis, B. queenslandensis, B. rattimassilensis, B. grahamii* | Europe, Asia, North America |
| *B. vinsonii* complex (e.g. GQ200861, GQ225708, GQ200857) | Dogs, Rodents | Rodent - associated genotypes (predominantly North American and Asian) with frequent spillover into dogs | North America, Europe, Asia, South America [11], Africa [12] |
| *B. washoensis* (FJ719016, AF050108) | Rodents, Rabbits | Rodent – associated genotypes (predominantly North American) | North America predominantly, Asia [13] |
| *GQ225707* | Rodent | *Rattus*-associated genotypes | Febrile Thai patient [14] |
| *HM116785* | Bats | European bat ectoparasite – associated genotypes | Polish forest worker [15] |

**References**

1. Breitschwerdt EB (2017) Bartonellosis, One Health and all creatures great and small. Vet Dermatol 28: 96-e21. Available: http://www.ncbi.nlm.nih.gov/pubmed/28133871%0Ahttp://doi.wiley.com/10.1111/vde.12413.

2. Mullins KE, Hang J, Jiang J, Leguia M, Kasper MR, et al. (2013) Molecular typing of “Candidatus *bartonella ancashi*,” A new human pathogen causing Verruga Peruana. J Clin Microbiol 51: 3865–3868. doi:10.1128/JCM.01226-13.

3. Flexman J, Lavis N, Kay I, Watson M, Metcalf C, et al. (1995) *Bartonella henselae* is a causative agent of cat scratch disease in Australia. J Infect 31: 241–245.

4. Lilley TM, Wilson CA, Bernard RF, Willcox E V., Vesterinen EJ, et al. (2017) Molecular Detection of Candidatus *Bartonella mayotimonensis* in North American Bats. Vector-Borne Zoonotic Dis 17: 243–246. Available: http://online.liebertpub.com/doi/10.1089/vbz.2016.2080.

5. Veikkolainen V, Vesterinen EJ, Lilley TM, Pulliainen AT (2014) Bats as reservoir hosts of human bacterial pathogen, *Bartonella mayotimonensis*. Emerg Infect Dis 20: 960–967. doi:10.3201/eid2006.130956.

6. Lin EY, Tsigrelis C, Baddour LM, Lepidi H, Rolain JM, et al. (2010) Candidatus *Bartonella mayotimonensis* and endocarditis. Emerg Infect Dis 16: 500–503. doi:10.3201/eid1603.081673.

7. Sangaré AK, Boutellis A, Drali R, Socolovschi C, Barker SC, et al. (2014) Detection of *Bartonella quintana* in African body and head lice. Am J Trop Med Hyg 91: 294–301. doi:10.4269/ajtmh.13-0707.

8. Woolley MW, Gordon DL, Wetherall BL (2007) Analysis of the first Australian strains of *Bartonella quintana* reveals unique genotypes. J Clin Microbiol 45: 2040–2043. doi:10.1128/JCM.00175-07.

9. Eremeeva M, Gerns H, Lydy S, Goo J, Ryan E, et al. (2007) Bacteremia, Fever, and Splenomegaly Caused by a Newly Recognized *Bartonella* Species. N Engl J Med 356: 2381–2387. doi:10.1056/NEJMoa065987.

10. Kabeya H, Colborn JM, Bai Y, Lerdthusnee K, Richardson JH, et al. (2010) Detection of *Bartonella tamiae* DNA in ectoparasites from rodents in Thailand and their sequence similarity with bacterial cultures from Thai patients. Vector-Borne Zoonotic Dis 10: 429–434. Available: http://www.ncbi.nlm.nih.gov/pubmed/20017718.

11. Fleischman DA, Chomel BB, Kasten RW, André MR, Goncąlves LR, et al. (2015) *Bartonella clarridgeiae* and *Bartonella vinsonii* subsp. *berkhoffii* exposure in captive wild canids in Brazil. Epidemiol Infect 143: 573–577. doi:10.1017/S0950268814001277.

12. Kernif T, Aissi M, Doumandji S-E, Chomel BB, Raoult D, et al. (2010) Molecular evidence of *Bartonella* infection in domestic dogs from Algeria, North Africa, by polymerase chain reaction (PCR). Am J Trop Med Hyg 83: 298–300. Available: http://www.pubmedcentral.nih.gov/articlerender.fcgi?artid=2911174&tool=pmcentrez&rendertype=abstract.

13. Sato S, Kabeya H, Miura T, Suzuki K, Bai Y, et al. (2012) Isolation and phylogenetic analysis of *Bartonella* species from wild carnivores of the suborder Caniformia in Japan. Vet Microbiol 161: 130–136. doi:10.1016/j.vetmic.2012.07.012.

14. Kosoy M, Bai Y, Sheff K, Morway C, Baggett H, et al. (2010) Identification of *Bartonella* infections in febrile human patients from Thailand and their potential animal reservoirs. Am J Trop Med Hyg 82: 1140–1145. doi:10.4269/ajtmh.2010.09-0778.

15. Podsiadly E, Chmielewski T, Karbowiak G, Kedra E, Tylewska-Wierzbanowska S (2010) The occurrence of spotted fever rickettsioses and other tick-borne infections in forest workers in Poland. Vector-Borne Zoonotic Dis 11: 985–989. Available: http://www.ncbi.nlm.nih.gov/pubmed/21083370.
