# Supplementary material for: Global fingerprint of humans on the distribution of Bartonella bacteria in mammals
Source: PLoS Negl Trop Dis. 2018 Nov 15;12(11):e0006865. doi: 10.1371/journal.pntd.0006865 (PMC6237287; doi:10.1371/journal.pntd.0006865)
Supplement: S1 Fig — The numbers in brackets indicate the lower and upper bounds of the 95% highest posterior density of the height of that branch, an estimate of the age of divergence of the node that the branch leads to. The width of the circle at each node is proportional to the Bayesian posterior probability support at that node. The blue tick mark numbers indicate the well-supported instances of Bartonella transferring between hosts that are used in inferring the timing of host transfers as detailed in S5 File. (PDF) [file pntd.0006865.s005.pdf]

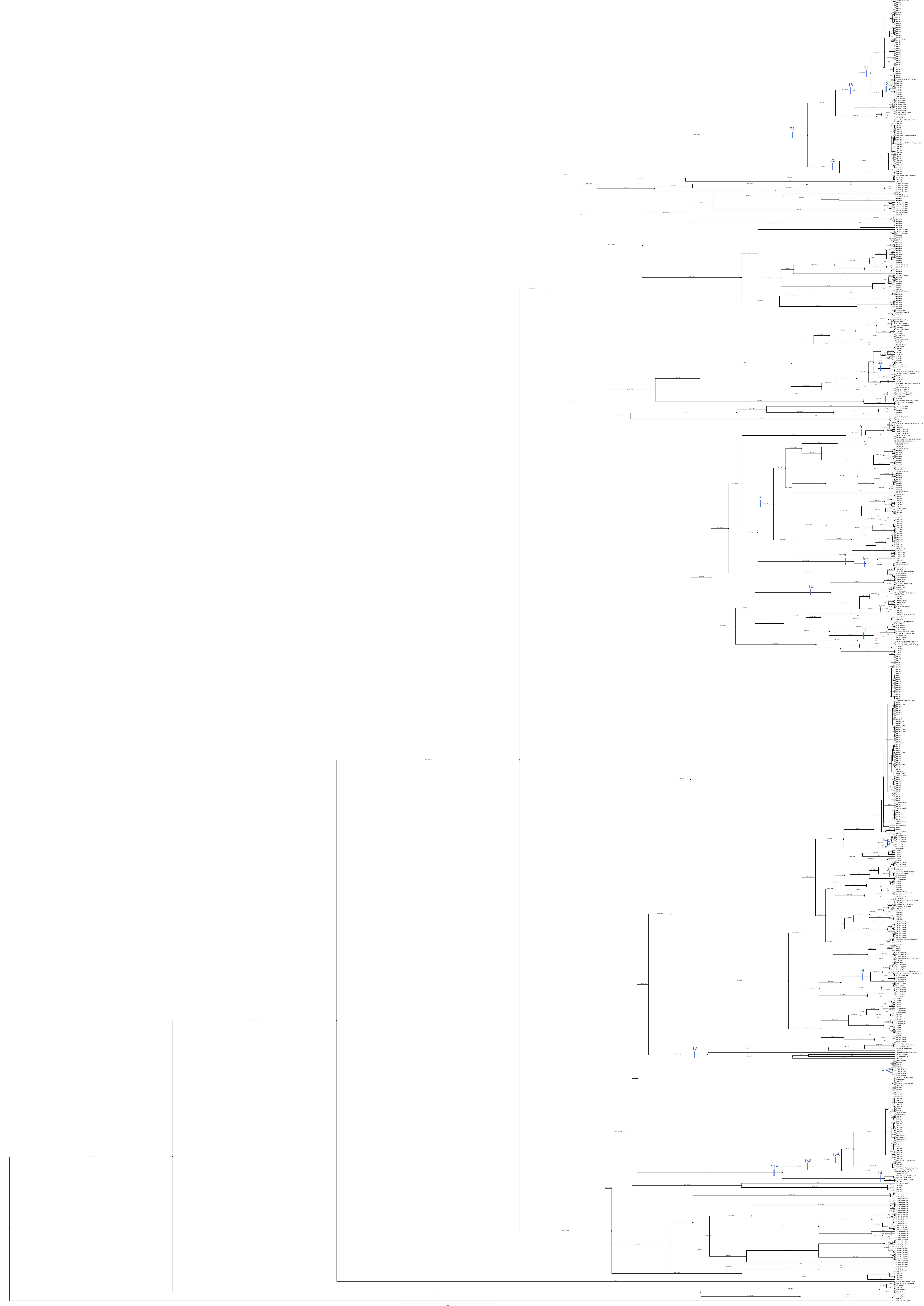

17
18
15
21
20
22
23
8
9
5
7
6
10
11
1
2
3
4
12
13
17A
16A
15A
14
