# Supplementary material for: Global fingerprint of humans on the distribution of Bartonella bacteria in mammals
Source: PLoS Negl Trop Dis. 2018 Nov 15;12(11):e0006865. doi: 10.1371/journal.pntd.0006865 (PMC6237287; doi:10.1371/journal.pntd.0006865)
Supplement: S2 Fig — The color of the tip label indicates the order of the host from which the genotype was derived (ectoparasites are colored according to their host). Highlighted tips indicate that multiple samples had the same sequence and the duplicate sequences can be found in S3 File by searching for the Genbank accession number. The number on each branch indicates the Bayesian posterior probability support leading to the node on the right. The icon at each tip indicates the geographic region from which the genotype was derived and the colored dot at each tip indicates the type of host it was isolate from, e.g. Rattus sp. rodents, non-Rattus rodents, wild animals, domestic animals. “SM” indicates a shrew or rodent (small mammal) as sometimes the actual host was not given and “Unk” indicates the continent from which the genotype was isolated is unknown. If a Bartonella species name is listed at a tip but not a Genbank accession number, the accession number is that of the sequence used in Judson et al. [16]. (PDF) [file pntd.0006865.s006.pdf]

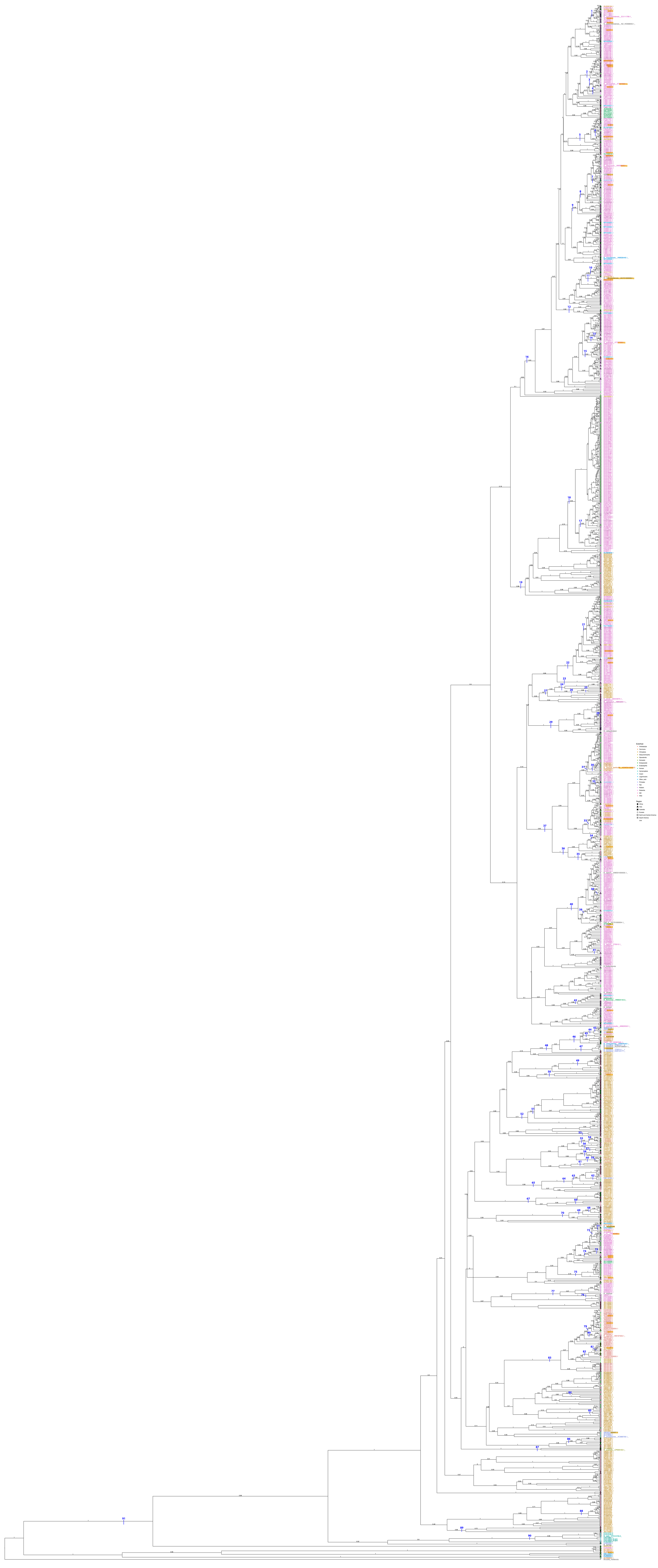
EctoHost
Artiodactyla
Carnivora
Chiroptera
Dasyuromorpha
Diprodontia
Domestic
Ectoparasite
Eulipotyphla
Human
Hymenoptera
Insect
Lagomorpha
Other_ecto
Primates
Rat
Rodent
Rodentia
SM
Wild
Region
Africa
Asia
Australia
Eurasia
North and Central America
South America
Unk
